# Characterization and phylogenetic analysis of the complete mitochondrial genome of *Mytella strigata* (Hanley 1843) (Bivalvia: Mytiloida: Mytilidae)

**DOI:** 10.1080/23802359.2021.1944382

**Published:** 2021-07-14

**Authors:** Chia-Hsuan Sung, Chih-Hsun Lin, Chang-Wen Huang, Liang-Jong Wang

**Affiliations:** a Planning and Information Division, Fisheries Research Institute, Keelung, Taiwan; b Department of Aquaculture, National Taiwan Ocean University, Keelung, Taiwan; c Mariculture Research Center, Fisheries Research Institute, Tainan, Taiwan; d Forest Protection Division, Taiwan Forestry Research Institute, Taipei, Taiwan

**Keywords:** Mitogenome, American brackish water mussel, *Mytella strigata*

## Abstract

We sequenced and assembled the complete mitochondrial genome (mitogenome) sequence of the American brackish water mussel *Mytella strigata*. The mitogenome, reaching 16,302 bp in length, includes 13 protein-coding genes, 2 ribosomal RNA genes, and 23 transfer RNA genes. The overall nucleotide composition of mitogenome was 25.17% A, 41.86% T, 11.83% C, and 21.13% G. The most common start and stop codons were GTG and TAA, respectively. The phylogenetic analysis based on mitogenomes showed that the families Mytilidae, Ostreidae, and Veneridae are a monophyletic group. The phylogenetic position of *M. strigata* is sister to *P. canaliculus* and *P. viridis*. In this study, mitogenomic sequence data will provide a better understanding for future studies of population genetics, biogeography, and pest surveillance of *M. strigata*.

The American brackish water mussel (*Mytella strigata*) is native to Central and South America from Guaymas, Sonora Mexico, Ecuador to the Galapagos Islands (Cardenas and Aranda 2000). Since 2014, its presence has been reported in Southeast Asia, including the Philippines (Michael et al. 2016), Singapore (Lim et al. 2018), Thailand (Sanpanich and Wells 2019), India (Jayachandran et al. 2019), and Taiwan. The species forms high-density fouling colonies attaching to concrete walls and drainage systems of clam ponds, boat hulls, and bottom sediment (Huang et al. 2021) using byssal threads to attach themselves to solid surfaces (Coyne et al. 1997). However, the mussels also bind to the shell of the hard clam disrupting normal feeding and breathing to clams in the pond. According to Lim et al. (2018), the external color of the shells was generally categorized into three groups: mostly bright green, orange to greenish-brown to gray; and uniformly black. The black variant is most commonly found in Taiwan. The special doubly uniparental inheritance was reported in *Mytella* (Alves et al. 2012; de Souza et al. 2015; Lim et al. 2018). The male mussels carry both matrilinear and patrilinear mtDNA and female mussels only carry the matrilinear mtDNA.

In this study, female *M. strigata* was collected from a hard clam brackish-water pond site in Yulin (32°46′36″N; 130°36′42″E), Taiwan, and stored in a Fisheries Research Institute in Keelung, Taiwan, with accession number FRIM10501 (contact person: CH Sung, chsung@mail.tfrin.gov.tw). Total genomic DNA was extracted from the foot of the mussel using the QIAamp DNA Mini Kit (QIAGEN) following the manufacturer’s instructions. The total DNA was sequenced using the Miseq sequencing platform (Illumina). The CLC Genomics Workbench V20 (QIAGEN) was used for sequencing reads quality analysis, reads trimming, and *de novo* assembling. The assembled mitogenome sequence was verified by the polymerase chain reaction (PCR) and Sanger approaches. PCR was amplified using three primer sets (Mst-L1F: 5′-TGTGGTTGAGCGAGGTGAA-3′, Mst-L1R: 5′-AATCAGACACCGCCTATTCG-3′, Mst-L2F: 5′-GGATGCGTATAAGCTGGATAGT-3′, Mst-L2R: 5′-AGGACCTAACATCTCTGGACAC-3′, Mst-L3F: 5′-TC TTGCTGGCGGAATCACTA-3′, and Mst-L3R: 5′-TCTGAGCATTAA GCATCTACGA-3′) with the following cycle: initial denaturation at 94 °C for 2 min, 35 cycles at 94 °C for 30 s, followed by annealing at 64 °C for 30 s, extension at 72 °C for 8 min, and a final extension at 72 °C for 10 min. The locations of the protein-coding genes, ribosomal RNAs (rRNAs), and transfer RNAs (tRNAs) were predicted by using MITOS Web Server (Bernt et al. 2013) and identified by alignment with other mitogenome sequence of Mytilidae mussel. The AT and GC skew was calculated according to the following formulas: AT skew = (A – T)/(A + T) and GC skew = (G – C)/(G + C) (Perna and Kocher 1995).

The complete DNA sequence of the *M. strigata* mitochondria reaching 16,302 bp in length (GenBank Accession No. MT991018) includes 13 protein-coding genes, 2 rRNA genes, and 23 tRNA genes. The overall nucleotide composition of mitogenome was 25.17% A, 41.86% T, 11.83% C, and 21.13% G. The AT and GC skewness of mitogenome sequence was −0.2490 and 0.2819, showing the T-skew and G-skew. The most common shared start codon between the 13 protein-coding genes was GTG (*atp6*, *cox2*, *nd1*, *nd4l*, *nd3*, *nd5*, *nd4*), followed by ATG (*cytb*, *cox3*, *cox1*), ATT (*nd6, atp8*), and ATA (*nd2*). The most common termination codons was TAA (*atp6*, *cytb*, *cox2*, *nd1*, *nd3*, *cox3*, *nd2*, *atp8*, *cox1*), followed by TAG (*nd4l*, *nd6*, *nd5*, *nd4*). The mitogenome of *M. strigata* contains 2 tRNA-Met genes, the same as most Mytilidae mussels.

We reconstructed the phylogenetic relationships of 23 Bivalvia species and the *Babylonia lutosa* as outgroup based on 12 protein-coding genes (excluding the atp8 gene) DNA sequences with maximum likelihood (ML) method (Figure 1). The clade including species attributing to Mytilidae was highly supported (100%). The families Mytilidae, Ostreidae, and Veneridae are a monophyletic group based on our result. The phylogenetic position of Mytilidae is sister to Ostreidae. The result is consistent with the previous study of the green-lipped mussel (Ranjard et al. 2018). The phylogenetic position of *M. strigata* is sister to the group of *P. canaliculus* (GMG766134) and *P. viridis* (NC_018362). The phylogenetic analysis based on DNA sequencing of *cox1* gene showed a close relationship between the Taiwan-acquired mussels and mussels from Singapore, India, and the Philippines (Huang et al. 2021). Our results shall provide a better understanding in the evolutionary histories of the Mytilidae and relative species. In this study, mitogenomic sequence data will provide useful information for future studies for population genetics, biogeography, and pest surveillance of *M. strigata*.

**Figure 1.**
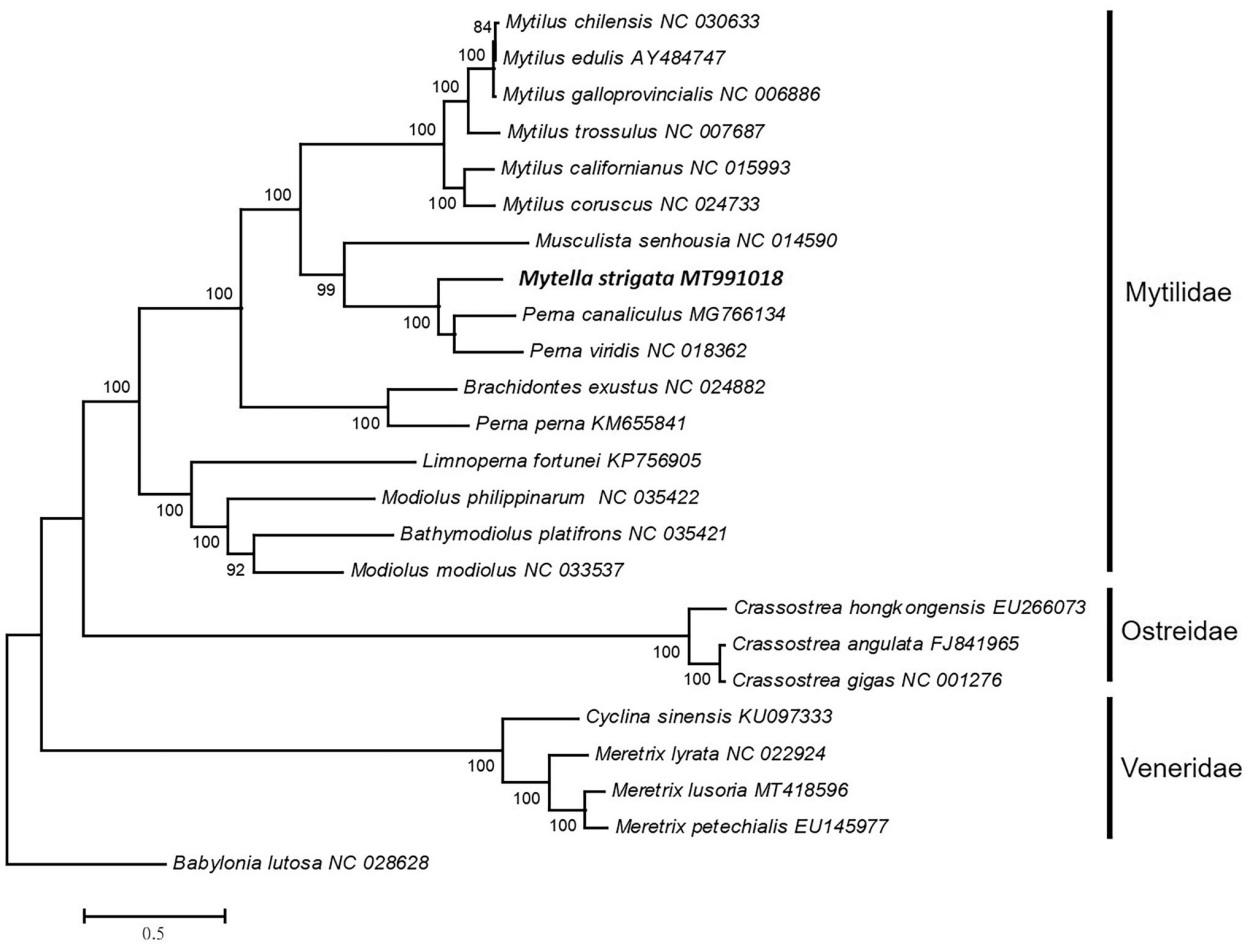
Phylogenetic tree of the 23 Bivalvia species based on the DNA sequence of 12 protein-coding genes. The tree was reconstructed with the maximum likelihood (ML) method using MEGA v.6 (Tamura et al. 2013) based on GTR + I + G model. Bootstrap values (1000 replications) greater than 70% are shown at the branch nodes.

## Data Availability

The genome sequence data that support the findings of this study are openly available in GenBank of NCBI at https://www.ncbi.nlm.nih.gov, accession number MT991018.
